# Supplementary figures and images for: A quasi-experimental study on stethoscopes contamination with multidrug-resistant bacteria: Its role as a vehicle of transmission
Source: PLoS One. 2021 Apr 22;16(4):e0250455. doi: 10.1371/journal.pone.0250455 (PMC8062016; doi:10.1371/journal.pone.0250455)

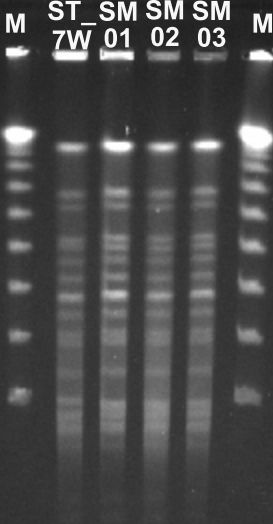

Supplement: S1 Fig — M, lambda ladder; ST_7W, K. pneumoniae from the stethoscope; SM 01 to 03, K. pneumoniae isolates form the patients. (PDF) [file pone.0250455.s001.pdf]
