# Supplementary material for: A quasi-experimental study on stethoscopes contamination with multidrug-resistant bacteria: Its role as a vehicle of transmission
Source: PLoS One. 2021 Apr 22;16(4):e0250455. doi: 10.1371/journal.pone.0250455 (PMC8062016; doi:10.1371/journal.pone.0250455)
Supplement: S1 File — (PDF) [file pone.0250455.s002.pdf]

First questionnaire

Study registration number: SC18OESI0120

Hello!
My name is Rae-Seok Lee, and I am a clinical lecturer at the department of infectious diseases at Yeouido St. Mary's Hospital. This questionnaire is part of a study planned to investigate the relationship between stethoscope cleaning by hospital healthcare personnel and multidrug-resistant organism contamination rate.
After you consent to participate and begin to complete the questionnaire, we will inoculate the medium by directly touching it with your stethoscope. The culture results will be analyzed by sex, age, job group, unit, and stethoscope cleaning habits. In addition, the same process will be repeated after providing an education about stethoscope cleaning to assess the changes in the contamination rate, changes in cleaning habits, and convenience of disinfection techniques.
Your data will be used anonymously and will not be disclosed to third parties.
We deeply appreciate your participation in the questionnaire.

General information

Question 1) What is your biological sex?
1) Male 2) Female

Question 2) What age group do you belong to?
1) 20-29 years 2) 30-39 years 3) 40-49 years 4) 50 years or older

Question 3) How long have you been using your stethoscope?
1) Under 6 months 2) 6 months to 1 year 3) More than 1 year

Question 4) What is your profession?
If you chose internal medicine, please answer question 4-1.
1) Physician (① Internal medicine ② Surgery ③ Neurosurgery ④ Emergency medicine ⑤ OB/Gyn/Pediatrics ⑥ Intern)
2) Nurse (① Medical ward ② Surgical ward ③ ICU ④ ED)

Question 4-1) What is your specialty?
1) Infectious disease 2) Endocrinology 3) Rheumatology 4) Gastroenterology 5) Cardiology
6) Nephrology 7) Pulmonology 8) Hematology-Oncology

Question 5) How long have you been working at the hospital?
1) Less than 2 years 2) 2−4.9 years 3) 5−9.9 years 4) 10 years or longer

Question 6) Do you use your own stethoscope when you examine a patient with MRDO requiring contact precautions?
1) Yes 2) No 3) Sometimes I use an available stethoscope

If you chose 1), please answer question 6-1.

Question 6-1) What is the reason for using your own stethoscope instead of an available stethoscope?
1) I had no idea there was an available stethoscope 2) The stethoscope available at the site is difficult to access
3) It's difficult to accurately auscultate with the available stethoscope

Questions about stethoscope cleaning

Question 1) Do you use your own stethoscope?
1) Yes 2) No

Question 2) How often do you clean your stethoscope?
1) After examining every patient 2) Once a day 3) Once a week to once a month
4) Sometimes 5) I don't clean it at all
If you chose 5, do not answer questions 3) and 4).

Question 3) How do you clean your stethoscope?
1) Ethanol based hand sanitizer 2) Alcohol swab
3) Soap and water

Question 4) Where do you usually clean your stethoscope?
1) Patient's bedside 2) Nurses' station 3) Outpatient clinic

Question 5) Have you been taught about the need to clean stethoscopes?
1) Yes 2) No
If you chose 2, do not answer question 6.

Question 6) If you have been taught, where were you taught?
1) School 2) Hospital 3) From a senior healthcare professional or colleagues

Question 7) Have you been taught about how to clean stethoscopes?
1) Yes 2) No
If you chose 2, do not answer question 8.

Question 8) If you have been taught, where were you taught?
1) School 2) Hospital 3) From a senior healthcare professional or colleagues

Questions about hand hygiene

Question 1) How many times do you wash your hands during your shift?
1) 5 times or fewer 2) 6−10 times 3) 11−20 times 4) 21−30 times 5) 31 times or

more

Question 2) How long do you wash your hands during your shift?

1) 10 seconds or less 2) 11−20 seconds 3) 21−30 seconds 4) 31–40 seconds 5) 41 seconds or longer

Question 3) Do you know exactly when you are required to wash your hands? (WHO-5 moments of hand hygiene)?
1) Yes 2) No

Questions about infection control awareness

Question 1) Do you think regular stethoscope cleaning by healthcare professionals is effective in preventing patient-patient infection?
1) I strongly agree 2) I agree 3) I don’t know 4) I disagree 5) I strongly disagree

Question 2) Do you know the types of MRDOs in hospitals that require contact precautions?
1) I know them well 2) I know some 3) I don’t know at all

Question 3) Do you think our hospital implements good infection control measures?
1) I strongly agree 2) I agree 3) I don’t know 4) I disagree 5) I strongly disagree

Question 4) Please provide any comments you have about the hospital’s infection control.

We deeply appreciate your time and participation.

Your data will be valuable to our research.

Thank you.
