# Supplementary material for: A quasi-experimental study on stethoscopes contamination with multidrug-resistant bacteria: Its role as a vehicle of transmission
Source: PLoS One. 2021 Apr 22;16(4):e0250455. doi: 10.1371/journal.pone.0250455 (PMC8062016; doi:10.1371/journal.pone.0250455)
Supplement: S2 File — (PDF) [file pone.0250455.s003.pdf]

# 1차 설문지

**연구등록번호 :** SC18OESI0120

안녕하십니까?
여의도 성모병원 감염내과 임상강사 이래석입니다, 본 설문지는 원내 의료진의 청진기 소독(stethoscope cleaning) 습관과 다제내성균 오염률의 관계를 보기 위한 연구를 위해 계획되었습니다.
연구에 동의 후 설문지 양식을 작성하여 주시는 동안에 귀하의 청진기를 배지에 직접 접촉하여 배양을 진행할 예정입니다. 배양 결과는 성별, 연령, 직군, 부서 및 청진기 소독 습관 별로 분리하여 분석할 예정입니다. 또한 청진기 소독에 대한 교육 후에 다시 동일한 과정을 반복하여 청진기 오염률과 소독습관의 변화, 소독 방법의 편의성 등을 평가할 예정입니다.
설문에 응해 주신 분들의 모든 자료는 익명화 작업되어 익명성이 보장되며 외부에 절대 노출되지 않습니다.
바쁘신 와중에 귀중한 시간을 내어 설문에 응해주셔서 진심으로 감사드립니다.

## 일반적 설문사항

질문1) 귀하의 성별은?
1) 남자 2) 여자

질문2) 귀하의 연령대는?
1) 20-29세 2) 30-39세 3) 40-49세 4) 50세 이상

질문3) 귀하가 사용하고 계시는 청진기의 사용기간은?
1) 6개월 미만 2) 6개월-1년 미만 3) 1년 이상

질문4) 귀하가 근무하시는 직군은?
**내과를 선택하신 경우 4-1 문항에 답해주시기 바랍니다.**
1) 의사 (① 내과 ② 외과 ③ 신경외과 ④ 응급의학과 ⑤ 산부인과/소아과 ⑥ 인턴 )
2) 간호사 (① 내과계병동 ② 외과계병동 ③ 중환자실 ④ 응급실)

질문4-1) 현재 근무 중인 내과의 분과는?
1) 감염내과 2) 내분비내과 3) 류마티스내과 4) 소화기내과 5) 순환기내과
6) 신장내과 7) 호흡기내과 8) 혈액종양내과

질문5) 병원 근무기간은 어떻게 되시나요?
1) 2년 미만 2) 2년 이상 -5년 미만 3) 5년 이상-10년 미만 4) 10년 이상

질문6) 접촉주의 다제내성균 보균 환자 진료시 본인 청진기를 사용하십니까?
1) 예 2) 아니오 3) 생각날 때만 가끔 비치된 청진기를 사용
**1)번을 선택하신 경우 6-1 문항에 답해주시기 바랍니다.**

질문6-1) 비치된 청진기가 아닌 본인 청진기 사용시 그 이유는 무엇입니까?
1) 비치되어 있는 줄 전혀 몰랐다 2) 비치되어 있는 청진기 접근성이 좋지 않다
3) 비치된 청진기로는 정확한 청진이 어렵다

## 청진기 소독(Stethoscope cleaning) 관련 설문사항

질문1) 귀하는 개인용 청진기를 사용하고 계십니까?
1) 예 2) 아니오

질문2) 귀하는 얼마나 자주 청진기를 소독하십니까?
1) 환자 한 명마다 2) 하루 한번 3) 일주일-한 달에 한번
4) 가끔 생각날 때 5) 전혀 소독하지 않는다
**5번을 택하신 경우 질문 3)-4)은 답하지 않습니다.**

질문3) 청진기 소독은 어떤 방법을 사용 하십니까?
1) 알콜젤 (Ethanol based hand sanitizer) 2) 알콜솜 (Alcohol swab)
3) 물과 비누 (soap and water)

질문4) 청진기 소독은 주로 어디에서 시행하십니까?
1) 환자 침상 옆 2) 병동 간호사실 3) 외래 진료실

질문5) 청진기 소독의 필요성에 대해 교육을 받은 적이 있습니까?
1) 네 2) 아니오
**2번을 택하신 경우 질문6 는 답하지 않습니다.**

질문6) 교육을 받은 적이 있다면 어디에서 받으셨습니까?
1) 학교 교육 2) 병원 내 교육 3) 선배 및 동료 의료진을 통해

질문7) 청진기 소독의 방법에 대해 교육을 받은 적이 있습니까?
1) 네 2) 아니오
**2번을 택하신 경우 질문8 은 답하지 않습니다.**

질문8) 교육을 받은 적이 있다면 어디에서 받으셨습니까?
1) 학교 교육 2) 병원 내 교육 3) 선배 및 동료 의료진을 통해

## 손위생 관련 설문사항

질문1) 평일 근무 시간 중 손을 씻는 횟수는 몇 회입니까?
1) 5회 이하 2) 6회-10회 3) 11회-20회 4) 21회-30회 5) 31회 이상

질문2) 근무 시간에 1회 손을 씻는 데 걸리는 시간은 얼마인가요?
1) 10초 이하 2) 11초-20초 3) 21초-30초 4) 31초-40초 5) 41초 이상

질문3) 손씻기는 어떠한 경우 (WHO-5가지 손위생 상황)에서 하는지 정확히 알고 계십니까?
1) 예 2) 아니오

## 감염관리 의식 설문사항

질문1) 의료진이 규칙적으로 청진기 소독을 한다면 환자와 환자 사이에 감염을 예방하는 데 효과가 있다고 생각하십니까?
1) 매우 그렇다 2) 대체로 그렇다 3) 잘 모르겠다 4) 대체로 그렇지 않다 5) 매우 그렇지 않다

질문2) 원내 접촉주의 다제내성균의 종류에 대해 알고 계십니까?
1) 잘 알고 있다 2) 일부는 알고 있다 3) 전혀 모른다

질문3) 우리 병원의 감염관리는 잘 이루어져 있다고 생각하십니까?
1) 매우 그렇다 2) 대체로 그렇다 3) 잘 모르겠다 4) 대체로 그렇지 않다 5) 매우 그렇지 않다

질문4) 원내 감염관리를 위해 하시고 싶은 말씀을 적어주세요.

| |
|---|
| |

귀한 시간을 내어 설문에 응하여 주셔서 진심으로 감사드립니다.

설문해 주신 자료는 귀중하게 사용하도록 하겠습니다.

고맙습니다.
