# Supplementary material for: A quasi-experimental study on stethoscopes contamination with multidrug-resistant bacteria: Its role as a vehicle of transmission
Source: PLoS One. 2021 Apr 22;16(4):e0250455. doi: 10.1371/journal.pone.0250455 (PMC8062016; doi:10.1371/journal.pone.0250455)
Supplement: S3 File — (PDF) [file pone.0250455.s004.pdf]

Second questionnaire

Study registration number: SC18OESI0120

Hello!
My name is Rae-Seok Lee, and I am a clinical lecturer at the department of infectious diseases at Yeouido St. Mary's Hospital. This questionnaire is part of a study planned to investigate the relationship between stethoscope cleaning habits by hospital healthcare personnel and multidrug-resistant organism contamination rate.
The purpose of this second questionnaire is to assess the effectiveness of and the compliance with the stethoscope cleaning education you have received, and we will compare the responses with that of the first questionnaires to analyze the effects of the education on you and other study groups. If you have lost or replaced your stethoscope after completing the first questionnaire for any reason, you will be excluded from the study.
Your data will be used anonymously and will not be disclosed to third parties.
We deeply appreciate your participation in the questionnaire.

General questions about stethoscope

Question 1) Have you lost or replaced your stethoscope since the first culture?
1) Yes 2) No

If you chose 1, you do not have to answer the questions below

Questions about stethoscope cleaning education

Question 1) Do you think the stethoscope cleaning education was helpful for changing your stethoscope cleaning habits?
1) Yes 2) No

If you chose 2, please answer Question 1-1.

Question 1-1) What is the reason for not changing your stethoscope cleaning habits even after the education?
1) I am not completely convinced of the need to clean 2) I forget often 3) I don't have time
4) Other:

Question 2) How often do you clean your stethoscope after receiving relevant training?
1) After examining every patient 2) Once a day 3) Once a week to once a month
4) Sometimes 5) I don't clean it at all

Question 3) How did you clean your stethoscope?
1) Ethanol based hand sanitizer 2) Alcohol swab

Question 4) Where did you usually clean your stethoscope?
1) Patient’s bedside 2) Nurses’ station 3) Outpatient clinic

Question 5) Would you clean your stethoscope more often if there were alcohol swabs available by patients’ bedside?
1) I strongly agree 2) I agree 3) I don’t know 4) I disagree 5) I strongly disagree

Question 6) What do you think is needed to improve the stethoscope cleaning rate?

Questions about changes in infection control awareness

Question 1) Do you think regular stethoscope cleaning by healthcare professionals is effective in preventing patient-patient infection?
1) I strongly agree 2) I agree 3) I don’t know 4) I disagree 5) I strongly disagree

Question 2) Do you know the types of MRDOs in hospitals that require contact precautions?
1) I know them well 2) I know some 3) I don’t know at all

Question 3) Do you think our hospital implements good infection control measures?
I strongly agree 2) I agree 3) I don’t know 4) I disagree 5) I strongly disagree

Question 4) Please provide any comments you have about the hospital’s infection control.

We deeply appreciate your time and participation.

Your data will be valuable to our research.

Thank you.
