# Supplementary material for: A quasi-experimental study on stethoscopes contamination with multidrug-resistant bacteria: Its role as a vehicle of transmission
Source: PLoS One. 2021 Apr 22;16(4):e0250455. doi: 10.1371/journal.pone.0250455 (PMC8062016; doi:10.1371/journal.pone.0250455)
Supplement: S4 File — (PDF) [file pone.0250455.s005.pdf]

2차 설문지

연구등록번호 : SC18OESI0120

안녕하십니까?
여의도 성모병원 감염내과 임상강사 이래석입니다, 본 설문지는 원내 의료진의 청진기 소독(stethoscope cleaning) 습관과 다제내성균 오염률의 관계를 보기 위한 연구를 위해 계획되었습니다.
2차 설문지는 귀하께서 받은 청진기 소독 교육의 효과 및 순응도를 평가하기 위한 것이며, 1차 설문지와 비교 분석하여 귀하 및 연구 집단별 효과 또한 분석할 예정입니다. 귀하께서 1차 설문지 이후 어떠한 이유로든 개인의 청진기를 분실 또는 교체하였을 경우 연구 대상 분석에서 제외될 예정입니다.
설문에 응해 주신 분들의 모든 자료는 익명화 작업되어 익명성이 보장되며 외부에 절대 노출되지 않습니다.
바쁘신 와중에 귀중한 시간을 내어 설문에 응해 주셔서 진심으로 감사드립니다.

청진기 관련 일반 설문사항

질문1) 1차 배양 후 현재까지 본인이 사용하던 청진기를 분실 또는 교체 하였습니까?
1) 네 2) 아니오

1)번을 택하신 경우 아래 질문에 답하지 않으셔도 됩니다.

청진기 소독(Stethoscope cleaning) 교육 효과 설문사항

질문1) 청진기 소독에 대한 교육이 귀하의 청진기 소독 습관 변화에 도움이 되었다고 생각하십니까?
1) 네 2) 아니오

2)번을 택하신 경우 질문1-1번에 답해 주시기 바랍니다.

질문1-1) 교육 이후에도 청진기 소독 습관 변화가 없는 이유는?
1) 필요성에 대한 공감 부족 2) 자주 잊게 됨 3) 시간 부족
4) 기타:

질문2) 교육과 홍보 후에 귀하는 얼마나 자주 청진기를 소독 하십니까?
1) 환자 한 명마다   2) 하루 한번   3) 일주일-한 달에 한번
4) 가끔 생각날 때   5) 전혀 소독하지 않는다

질문3) 청진기 소독은 어떤 방법을 주로 사용 하셨습니까?
1) 알콜젤 (Ethanol based hand sanitizer) 2) 알콜솜 (Alcohol swab)

질문4) 청진기 소독은 주로 어디에서 시행 하였습니까?
1) 환자 침상 옆   2) 병동 간호사실   3) 외래 진료실

질문5) 환자 침상에 일회용 알콜솜(alcohol swab)이 있다면 소독을 더 자주 하시겠습니까?
1) 매우 그렇다 2) 대체로 그렇다 3) 잘 모르겠다 4) 대체로 그렇지 않다 5) 매우 그렇지 않다

질문6) 청진기 소독률 향상을 위해 더 필요한 것은 무엇이라고 생각합니까?

감염관리 의식 변화 설문사항

질문1) 의료진이 규칙적으로 청진기 소독을 한다면 환자와 환자 사이에 감염을 예방하는 데 효과가 있다고 생각하십니까?
1) 매우 그렇다 2) 대체로 그렇다 3) 잘 모르겠다 4) 대체로 그렇지 않다 5) 매우 그렇지 않다

질문2) 원내 접촉주의 다제내성균의 종류에 대해 알고 계십니까?

1) 잘 알고 있다   2) 일부는 알고 있다   3) 전혀 모른다

질문3) 우리 병원의 감염관리는 잘 이루어져 있다고 생각하십니까?
1) 매우 그렇다 2) 대체로 그렇다 3) 잘 모르겠다 4) 대체로 그렇지 않다 5) 매우 그렇지 않다

질문4) 원내 감염관리를 위해 하시고 싶은 말씀을 적어주세요.

귀한 시간을 내어 설문에 응하여 주셔서 진심으로 감사드립니다.

설문해 주신 자료는 귀중하게 사용하도록 하겠습니다.

고맙습니다.
