# Supplementary material for: A quasi-experimental study on stethoscopes contamination with multidrug-resistant bacteria: Its role as a vehicle of transmission
Source: PLoS One. 2021 Apr 22;16(4):e0250455. doi: 10.1371/journal.pone.0250455 (PMC8062016; doi:10.1371/journal.pone.0250455)
Supplement: Attachment — Submitted filename: PLOS one_Response to Reviewer.docx [file pone.0250455.s006.docx]

**Reply to reviewer’s comments : Manuscript [PONE-D-20-26077]**

We would like to thank editor and reviewers for the invaluable comments and advices that help a great deal in improving our original manuscript. Please find attached our revised paper and below a summary of how we responded to the comments.

**Reviewer #1:** This is a well-conducted study regarding the effect of an educational program as an intervention to decrease the contamination rate of stethoscopes in use within a hospital. However, in this regard I have some observations:

1. Although the program was shown to have a positive effect on cleaning habits and cleaning frequency in stethoscopes, no effect on the contamination rate was observed. The authors focus the discussion of this point on aspects related to the training program; however, they do not consider the nature of the disinfectant used, which could also have an effect on the contamination rate. The disinfectant reported by the participants was ethyl alcohol, which has a good effect on bacteria, fungi and even viruses; however, it evaporates quickly, allowing re-contamination of the stethoscope in the time after cleaning, where the stethoscope can be in contact with the patient's environment or the patient himself.

***Response: We strongly agree that it is very challenging to use and recommend the alcohol-based disinfection methods due to its limitation as you mentioned. The manuscript text was revised to indicate this limitation in discussion. Please see lines 266-270.***

2. The authors make the following statement in the discussion: “This is the first study to evaluate the contamination rate of stethoscopes by nosocomial pathogens and MDR bacteria and to demonstrate the possibility of stethoscope as a vehicle for MDR bacteria such as CPE, but with several limitations. " This statement is not entirely correct and in any case, they must show evidence to support such statement.

***Response: The manuscript text was clarified to indicate that this is the first study to evaluate the contamination rate of MDR bacteria and to demonstrate the possibility of stethoscope as a vehicle for MDR bacteria. As you mentioned, this is not the first study to evaluate the contamination rate of stethoscope by nosocomial pathogen. Please, see lines 278-280. The originality of this results is already mentioned in the manuscript. This is described on lines 14-201 in introduction and lines 227-236 in discussion of the manuscript.***

3. In table 3 the names of the isolated bacteria must be written according to the binomial system, following the nomenclature rules for publication in scientific journals.

***Response: We appreciated your detailed comment. We corrected that points in the Table 3.***

**Reviewer #2:** This is an extremely well written article in a challenging area of research that has never been published before. They evaluated the potential for the stethoscope to transmit multidrug resistant (MDR) organisms, as well as the impact of education on stethoscope hygiene. While the latter has previously been shown to have little impact (and is consistent with the authors findings), the former is uniquely important, as it clearly demonstrate the potential for the stethoscope to serve as a MDR organism vector. This paper will be frequently cited, and will serve as an important contribution to the literature. I have reviewed over 1000 articles for various journals (including PLOS ONE) in my career. I have NEVER recommended acceptance without a number of suggestions. The only suggestion I have for this manuscript is that they suggest methods that may decrease the potential for MDR transmission by the stethoscope. There are a number of stethoscope hygiene strategies on the market that include barriers, caps, UV light, and disposable stethoscopes (although these are terrible tools) that do not require health care practitioner cleaning (which has been repetitively proven to be an ineffective intervention). It would be reasonable to suggest them here. Further, it is a covid world, and the potential for the stethoscope being a vector may have greater impact than when this reasearch started. Here are a few citations. Thank you for the privilege of reviewing this excellent manuscript. New Scope for the Stethoscope. Kalra S, Reddy S. Mayo Clin Proc Innov Qual Outcomes. 2020 Feb 5;4(1):1-2. Aseptic Barriers Allow a Clean Contact for Contaminated Stethoscope Diaphragms. Vasudevan R, Shin JH, Chopyk J, Peacock WF, Torriani FJ, Maisel AS, Pride DT. Mayo Clin Proc Innov Qual Outcomes. 2020 Feb 5;4(1):21-30. The stethoscope: a potential vector for COVID-19? Vasudevan RS, Bin Thani K, Aljawder D, Maisel S, Maisel AS. Eur Heart J. 2020 Sep 21;41(36):3393-3395. Comparing the auscultatory accuracy of health care professionals using three different brands of stethoscopes on a simulator. Mehmood M, Abu Grara HL, Stewart JS, Khasawneh FA. Med Devices (Auckl). 2014 Aug 14;7:273-81.

***Response: Thank you for your comment and suggestion. We are totally agree with your opinion. It will be important to comment other options. However, in this study, we did not provide other options to disinfect the stethoscopes rather we maintained conventional disinfection method as we focused to see habit change. The manuscript text was added to indicate this limitation in discussion. Also, we added new references in this revised manuscript. Please see lines 290-293.***

**Reviewer #3:** I just congratulate all the authors for this interesting study.
Line 22: please include a paragraph that describe HCWs stethoscope disinfection practice of HCWs from previously conducted studies and the challenges behind/ or the driving force/. Use the following article as an example and many others from PubMed
Ex:" Sahiledengle B. Stethoscope disinfection is rarely done in Ethiopia: What are the associatedfactors?PLoSONE.2019: 14(6):e0208365.

***Response: Thank you for this reviewing and comment. We agree to include detailed information about disinfection practice based on the relevant literature in introduction. The manuscript text was revised to indicate this information and we have added a references. About the challenges behind we thought was described in introduction. Please see lines 22-26.***

Line 36: Does the authors included all HCWs from the specific dep't ?

***Response: The manuscript has been edited to provide information on specific target population. Healthcare workers working in the general wards, intensive care units and emergency room, only those who voluntarily agreed to participate in this study were enrolled.. Please see lines 38-39.***

Line 39: self-administered or what ? what about the validity and reliability of the data collection tool ?

***Response: The questionnaire was developed by reviewing previous literatures and adapted to the Korean medical situation through investigator meeting. To improve the quality and consistency, we also pre-tested by three doctors and two nurses who did not participate in the study. Additional explanation was added to the manuscript, lines 54-57.***

line 45: "conducting a training for a month" is not clear: does the training given for 30 days? or how many days; how many hours ? when ? should be clear

***Response: The manuscript was revised more clearly about the frequency and time of training and education. Detailed information on the duration is presented in the figure 1 also. Please see lines 43-47.***

Line 95: We did not see a multivariate logistic regression table in the manuscript ?

***Response: Multivariate regression analysis was performed to evaluate the independent factors for contamination with nosocomial pathogens. However, that was not the main aim of this study, so we did not make a table or figure rather we described the result in the results lines 193-197.***
